# On Bandwidth Characteristics of Tuning Fork Micro-Gyroscope with Mechanically Coupled Sense Mode

**DOI:** 10.3390/s140713024

**Published:** 2014-07-21

**Authors:** Yunfang Ni, Hongsheng Li, Libin Huang, Xukai Ding, Haipeng Wang

**Affiliations:** 1 School of Instrument Science and Engineering, Southeast University, Nanjing 210096, China; E-Mails: niyunfang@126.com (Y.N.); huanglibin@seu.edu.cn (L.H.); dingxukai@126.com (X.D.); fanfankaka123@126.com (H.W.); 2 Key Laboratory of Micro-Inertial Instrument and Advanced Navigation Technology of Ministry of Education, Southeast University, Nanjing 210096, China

**Keywords:** MEMS, tuning fork, micro-gyroscope, bandwidth, mechanical coupling

## Abstract

The bandwidth characteristics of a tuning fork micro-gyroscope with mechanically coupled sense mode were investigated in this paper to provide some references for mechanical bandwidth design. The concept of sense mode mechanical coupling is introduced first. Theoretical frequency response analyses were then carried out on the mechanical part of the gyroscope. Equations representing the relationships between the differential output signal and the frequency of the input angular rate were deduced in full frequency range and further simplified in low frequency range. Based on these equations, bandwidth characteristics under ideal and non-ideal conditions are discussed. Analytical results show that under ideal conditions, the bandwidth characteristics of a tuning fork micro-gyroscope are similar to those of a single mass micro-gyroscope, but under non-ideal conditions, especially when sense mass and/or stiffness are asymmetric, the bandwidth characteristics would be quite different because the in-phase mode would participate in the anti-phase vibration response. Experimental verifications were carried out on two micro-gyroscope prototypes designed in our laboratory. The deduced equations and analytical results can be used in guiding the mechanical bandwidth design of tuning fork micro-gyroscopes with mechanically coupled sense mode.

## 1. Introduction

Micro-gyroscopes have achieved a rapid development in the past several decades. In comparison with their conventional counterparts, micro-gyroscopes hold the advantages of small size, low cost, reduced power consumption and batch fabrication, *etc*. With gradually mature design and manufacturing technologies, they are now widely used in various fields, such as the automotive industry, consumer electronics, aerospace navigation and military weapons.

According to the number of Coriolis masses, micro-gyroscopes can be classified into three types: single mass gyroscopes, tuning fork gyroscopes and gyroscope arrays. Nowadays tuning fork gyroscopes are becoming the most popular ones. They are less complicated than gyroscope arrays and more attractive than the single mass ones because of their inherent common mode rejection capability.

A tuning fork micro-gyroscope is generally composed of two identical single mass structures. Nevertheless, diverse configuration patterns have been implemented. Analog Devices Inc. configured its commercialized ADXRS tuning fork micro-gyroscope with two identical single mass structures which are mechanically independent with each other [1]. LITEF GmbH and Middle East Technical University configured their tuning fork micro-gyroscopes with two identical single mass structures which are mechanically coupled in drive mode, but mechanically independent in sense mode [2,3]. Moreover, the Georgia Institute of Technology, University of California—Irvine and Robert Bosch GmbH have configured their tuning fork micro-gyroscopes with two identical single mass structures which are mechanically coupled in both drive mode and sense mode [4–6].

The introduction of mechanical coupling can improve the matching extent of the two single mass structures by one or two orders of magnitude. That can enhance the tuning fork effect in common mode rejection [7,8]. Meanwhile, the anti-phase vibration mode of the mechanically coupled structures can achieve a much higher Q-factor than that of the mechanically independent structures under the same vacuum conditions [9]. A high Q-factor in drive mode can reduce the drive voltages, electrical parasitic signals and the sensor power consumption. A high Q-factor in sense mode can improve the resolution and bias stability of the gyroscope. Therefore, mechanical coupling between the two single mass structures benefits the overall performance of a tuning fork micro-gyroscope.

Nevertheless, mechanical coupling also has some potential drawbacks. For a tuning fork micro-gyroscope with mechanically coupled single mass structures, an in-phase vibration mode always coexists with the anti-phase one. This in-phase vibration mode is susceptible to linear environmental vibrations [10]. In the presence of structural asymmetry, it would even participate in anti-phase load induced vibration responses. Consequently, if its natural frequency is not appropriately configured, the in-phase mode would interfere with the gyroscope output. Bandwidth characteristics would also be affected in such a case.

In practice, the angular rate signal is generally time dependent and contains a series of frequency components. Therefore, a micro-gyroscope should have a certain bandwidth for dynamic angular rate detection. So far, the bandwidth characteristics of single mass micro-gyroscopes are quite familiar, but these of tuning fork micro-gyroscopes are not reported in detail yet. For a tuning fork micro-gyroscope with mechanically independent single mass structures, the bandwidth characteristics are similar to those of two single mass micro-gyroscopes. For a tuning fork micro-gyroscope with mechanically coupled single mass structures, the bandwidth characteristics are more complicated and deserve further study.

This paper presents a study on the bandwidth characteristics of tuning fork micro-gyroscopes with mechanically coupled sense mode. The relationship between the differential output of the sense mode vibration response and the frequency of the input angular rate was discussed under ideal (*i.e.*, symmetric) and non-ideal (*i.e.*, asymmetric) conditions. The specific influences of the in-phase mode on bandwidth characteristics under several non-ideal conditions were investigated. Conclusions were made to provide some references for the mechanical bandwidth design of tuning fork micro-gyroscopes.

The rest of this paper is organized as follows: Section 2 introduces the concept and working principle of a tuning fork micro-gyroscope with mechanically coupled sense mode. The coupling methods in sense direction were discussed at the same time. Section 3 describes the theoretical frequency response analyses. The equations representing the bandwidth characteristics were deduced in full frequency range, and further simplification was made in low frequency range. Section 4 discusses the bandwidth characteristics under ideal conditions. Section 5 discusses the bandwidth characteristics under non-ideal conditions. Three kinds of asymmetry were covered: asymmetric drive amplitude, asymmetric sense mass and asymmetric sense stiffness. Section 6 provides the experimental verification on two prototypes of tuning fork micro-gyroscope designed in our laboratory. The mechanical structure and the testing method were both introduced, and the correctness of the analytical results was verified. Section 7 concludes the whole paper.

## 2. Tuning Fork Micro-Gyroscope with Mechanically Coupled Sense Mode

**Figure 1.**
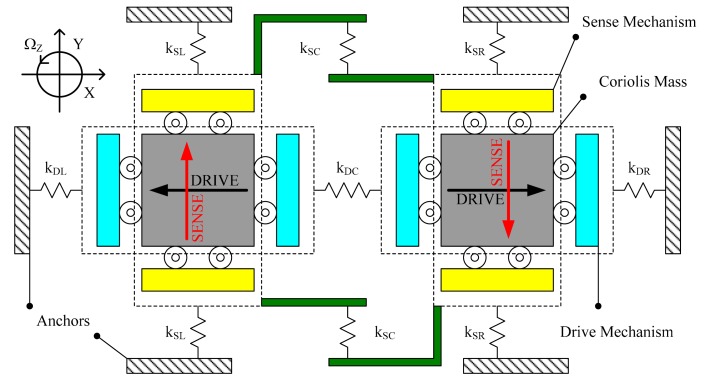
Schematic of a tuning fork micro-gyroscope with mechanically coupled drive mode and sense mode.

The conceptual schematic of a tuning fork micro-gyroscope with mechanically coupled sense mode is shown in Figure 1. The drive mode was also considered as mechanically coupled. As shown in Figure 1, while drive stiffness k_DL_ and k_DR_ exist between the drive masses and the anchors, a coupling stiffness k_DC_ is designed between the two drive masses to realize the drive mode mechanical coupling. Similarly, while sense stiffness k_SL_ and k_SR_ exist between the sense masses and the anchors, a coupling stiffness k_SC_ is designed between the two sense masses to realize the sense mode mechanical coupling. In-phase mode and anti-phase mode coexist both in drive direction and in sense direction.

In operation, the two drive masses would be actuated into anti-phase resonance vibration with the same amplitude in the drive direction (X-axis). When an input angular rate Ω_Z_ (Z-axis) was applied, due to the Coriolis effect, the two Coriolis masses would encounter anti-phase Coriolis forces in the sense direction (Y-axis). Hence, the two sense masses would be forced into anti-phase vibration in the sense direction with the same rate-related amplitude. A differential output can be obtained indicating Ω_Z_.

Generally, the two single mass structures of a tuning fork micro-gyroscope share the same center line in the drive direction but occupy two parallel center lines in the sense direction. Therefore, mechanical coupling in sense mode is much more difficult to realize than that in drive mode.

**Figure 2.**
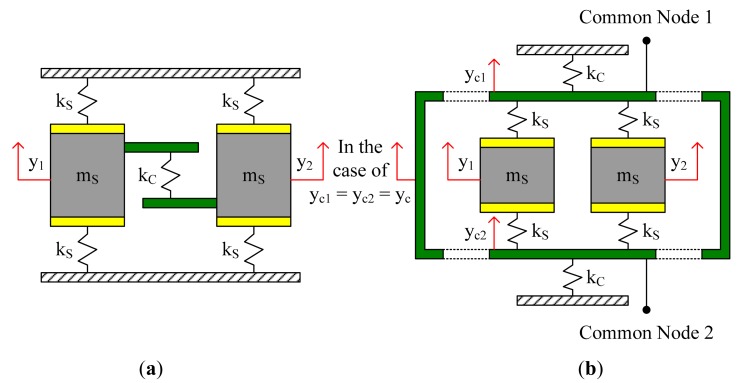
(**a**) Type-A of sense mode coupling method; (**b**) Type-B of sense mode coupling method.

Two main kinds of sense mode coupling methods are shown in Figure 2. Coupling method Type-A is similar to that shown in Figure 1. While sense stiffness k_S_ exist between the sense masses and the anchors, a coupling stiffness k_C_ is introduced between the two sense masses. In coupling method Type-B, there are no direct connections between the sense masses and the anchors. Each sense mass is linked to the common nodes with sense stiffness k_S_. Two coupling stiffnesses k_C_ are introduced between the common nodes and the anchors. Mechanical coupling is realized with the combination of k_S_, k_C_ and the common nodes.

Under ideal conditions, with coupling method Type-A, the natural frequencies of the in-phase mode and the anti-phase mode in sense direction would be:

(1) $$\begin{array}{cc} \omega_{in-\text{phase}}=\sqrt{\frac{2k_{S}}{m_{S}}}, & \omega_{\text{anti}-\text{phase}}=\sqrt{\frac{2k_{S}+2k_{C}}{m_{S}}} \end{array}$$

With coupling method Type-B, ignoring the small mass of the common nodes, the natural frequencies of the in-phase mode and the anti-phase mode in sense direction would be:

(2) $$\begin{array}{cc} \omega_{in-\text{phase}}=\sqrt{\frac{2k_{S}-\frac{4{k_{S}}^{2}}{2k_{S}+k_{C}}}{m_{S}}}, & \omega_{\text{anti}-\text{phase}}=\sqrt{\frac{2k_{S}}{m_{S}}} \end{array}$$

It can be found that the natural frequency of the in-phase mode is lower than that of the anti-phase mode. In coupling method Type-A, the frequency difference between these two modes can be adjusted by k_C_. In coupling method Type-B, the frequency difference can be adjusted by k_S_ and/or k_C_. Unless via designing specialized mechanical coupling components which show different elastic characteristics in in-phase motion and in anti-phase motion [5,8], it is difficult to make the natural frequency of the in-phase mode to be higher than that of the anti-phase mode.

Coupling method Type-A is easy to realize in structure design, but it has the disadvantage of torque unbalance because the coupling stiffness k_C_ only exists at one side of each sense mass. Coupling method Type-B is more difficult in structure design, but its vibration stability is better than that of Type-A. Moreover, in Type-B, there are no direct connections between the sense masses and the anchors, and the natural frequency of the anti-phase mode is independent of k_C_ according to Equation (2), hence the anti-phase vibration is less susceptible to the detrimental stress and strain passed through the anchors from the substrate and the package. For the tuning fork micro-gyroscope designed in our laboratory, coupling method Type-B was employed in sense mode.

## 3. Theoretical Frequency Response Analysis

### 3.1. Complete Equation in Full Frequency Range

In order to study the bandwidth characteristics of a tuning fork micro-gyroscope with mechanically coupled sense mode, theoretical frequency response analysis was carried out to get the relationship between the differential output signal and the frequency of the input angular rate.

A time dependent angular rate signal contains a series of frequency components. Without loss of generality, we defined the input angular rate as:

(3) $$\Omega_{Z}\left(t\right)=\Omega \cos\left(\omega t\right)$$

where Ω is the amplitude and ω is the frequency of the input angular rate.

The two drive masses are usually actuated into anti-phase resonance vibration with constant amplitudes. Therefore, drive velocity can be defined as:

(4) $$\begin{array}{cc} {ẋ}_{1}\left(t\right)=-A_{d1}\omega_{d}\sin\left(\omega_{d}t\right), & {ẋ}_{2}\left(t\right)=+A_{d2}\omega_{d}\sin\left(\omega_{d}t\right) \end{array}$$

where A_d1_ and A_d2_ represent the drive amplitudes of the two drive masses, ω_d_ is the natural frequency of the anti-phase mode in drive direction, which is hereinafter referred as drive frequency. It's assumed that each sense mass is approximately equal to each Coriolis mass. With m_1_ and m_2_ denoting the two Coriolis masses, the two Coriolis forces in the sense direction can be expressed as:

(5) $$F_{c1}\left(t\right)=-2m_{1}\Omega_{Z}\left(t\right){ẋ}_{1}\left(t\right)=+m_{1}A_{d1}\omega_{d}\Omega \left[\sin\left(\left(\omega_{d}+\omega \right)t\right)+\sin\left(\left(\omega_{d}-\omega \right)t\right)\right]$$

(6) $$F_{c2}\left(t\right)=-2m_{2}\Omega_{Z}\left(t\right){ẋ}_{2}\left(t\right)=-m_{2}A_{d2}\omega_{d}\Omega \left[\sin\left(\left(\omega_{d}+\omega \right)t\right)+\sin\left(\left(\omega_{d}-\omega \right)t\right)\right]$$

It can be found that two frequency components, ω_d_+ω and ω_d_−ω, coexist in the Coriolis forces. If we define:

(7) $$\begin{array}{cc} F_{R}\left(t\right)=\omega_{d}\Omega \sin\left(\left(\omega_{d}+\omega \right)t\right), & F_{L}\left(t\right)=\omega_{d}\Omega \sin\left(\left(\omega_{d}-\omega \right)t\right) \end{array}$$

then the dynamic force vector applied to the sense mode vibration system can be expressed as:

(8) $$\text{F}=\left[\begin{array}{c} F_{c1}\left(t\right)\\ F_{c2}\left(t\right) \end{array}\right]=\left[\begin{array}{c} +m_{1}A_{d1}\left(F_{R}\left(t\right)+F_{L}\left(t\right)\right)\\ -m_{2}A_{d2}\left(F_{R}\left(t\right)+F_{L}\left(t\right)\right) \end{array}\right]$$

No matter what kind of structure a tuning fork micro-gyroscope has, the coupled dynamic equation of its undamped free vibration in the 2-DOF sense mode vibration system can always be expressed as:

(9) $$\left[\begin{array}{cc} m_{1} & 0\\ 0 & m_{2} \end{array}\right]\left[\begin{array}{c} {\ddot{y}}_{1}\\ {\ddot{y}}_{2} \end{array}\right]+\left[\begin{array}{cc} k_{11} & k_{12}\\ k_{12} & k_{22} \end{array}\right]\left[\begin{array}{c} y_{1}\\ y_{2} \end{array}\right]=\left[\begin{array}{c} 0\\ 0 \end{array}\right]$$

where k_11_ and k_22_ is the effective stiffness corresponding to the sense displacement y_1_ and y_2_, k_12_ is the coupling stiffness between y_1_ and y_2_. Natural frequencies ω_1_, ω_2_ and their corresponding modal vectors **Y**^(1)^, **Y**^(2)^ can be calculated from Equation (9):

(10) $$\begin{array}{cc} {\omega_{1}}^{2}=\frac{k_{22}m_{1}+k_{11}m_{2}}{2m_{1}m_{2}}-\sqrt{{\left(\frac{k_{22}m_{1}+k_{11}m_{2}}{2m_{1}m_{2}}\right)}^{2}+\frac{{k_{12}}^{2}-k_{11}k_{22}}{m_{1}m_{2}}}, & {\text{Y}}^{\left(1\right)}=\left[\begin{array}{c} {Y_{1}}^{\left(1\right)}\\ {Y_{2}}^{\left(1\right)} \end{array}\right]=\left[\begin{array}{c} {Y_{1}}^{\left(1\right)}\\ r_{1}{Y_{1}}^{\left(1\right)} \end{array}\right],r_{1}=-\frac{k_{11}-m_{1}{\omega_{1}}^{2}}{k_{12}} \end{array}$$

(11) $$\begin{array}{cc} {\omega_{2}}^{2}=\frac{k_{22}m_{1}+k_{11}m_{2}}{2m_{1}m_{2}}+\sqrt{{\left(\frac{k_{22}m_{1}+k_{11}m_{2}}{2m_{1}m_{2}}\right)}^{2}+\frac{{k_{12}}^{2}-k_{11}k_{22}}{m_{1}m_{2}}}, & {\text{Y}}^{\left(2\right)}=\left[\begin{array}{c} {Y_{1}}^{\left(2\right)}\\ {Y_{2}}^{\left(2\right)} \end{array}\right]=\left[\begin{array}{c} {Y_{1}}^{\left(2\right)}\\ r_{2}{Y_{1}}^{\left(2\right)} \end{array}\right],r_{2}=-\frac{k_{12}}{k_{22}-m_{2}{\omega_{2}}^{2}} \end{array}$$

where r_1_, r_2_ are the amplitude ratios of the two sense masses in the modal vectors.

Mode superposition method, a common method in mechanical vibration theories to obtain the dynamic response of a linear multi-DOF vibration system using the natural frequencies and modal vectors, was employed to get the vibration response of the sense mode vibration system under the action of the force vector shown in Equation (8). Considering modal damping ratios, the vibration response can be calculated from the following decoupled dynamic equations:

(12) $${\ddot{p}}_{1}\left(t\right)+2\xi_{1}\omega_{1}{ṗ}_{1}\left(t\right)+{\omega_{1}}^{2}p_{1}\left(t\right)=P_{1}\left(t\right)$$

(13) $${\ddot{p}}_{2}\left(t\right)+2\xi_{2}\omega_{2}{ṗ}_{2}\left(t\right)+{\omega_{2}}^{2}p_{2}\left(t\right)=P_{2}\left(t\right)$$

where ξ_1_, ξ_2_ are the modal damping ratios corresponding to the two modal vectors, p_1_(t), p_2_(t) are the generalized coordinates and P_1_(t), P_2_(t) are the generalized forces. The relationship between the actual coordinates y_1_(t), y_2_(t) and the generalized ones, along with the relationship between the actual forces F_c1_(t), F_c2_(t) and the generalized ones, are shown as follows:

(14) $$\left[\begin{array}{c} y_{1}\left(t\right)\\ y_{2}\left(t\right) \end{array}\right]=\left[\begin{array}{cc} \frac{1}{\sqrt{m_{1}+{r_{1}}^{2}m_{2}}} & \frac{1}{\sqrt{m_{1}+{r_{2}}^{2}m_{2}}}\\ \frac{r_{1}}{\sqrt{m_{1}+{r_{1}}^{2}m_{2}}} & \frac{r_{2}}{\sqrt{m_{1}+{r_{2}}^{2}m_{2}}} \end{array}\right]\left[\begin{array}{c} p_{1}\left(t\right)\\ p_{2}\left(t\right) \end{array}\right],\left[\begin{array}{c} P_{1}\left(t\right)\\ P_{2}\left(t\right) \end{array}\right]=\left[\begin{array}{cc} \frac{1}{\sqrt{m_{1}+{r_{1}}^{2}m_{2}}} & \frac{r_{1}}{\sqrt{m_{1}+{r_{1}}^{2}m_{2}}}\\ \frac{1}{\sqrt{m_{1}+{r_{2}}^{2}m_{2}}} & \frac{r_{2}}{\sqrt{m_{1}+{r_{2}}^{2}m_{2}}} \end{array}\right]\left[\begin{array}{c} F_{c1}\left(t\right)\\ F_{c2}\left(t\right) \end{array}\right]$$

With Equations (12)–(14), the differential output of the sense mode vibration response can be obtained as:

(15) $$y_{o}\left(t\right)=y_{1}\left(t\right)-y_{2}\left(t\right)=\left(E_{1}Y_{\omega 1}+E_{2}Y_{\omega 2}\right)\Omega$$

where Y_ω1_ represents the response portion related to the 1st order modal parameters ω_1_ and r_1_, Y_ω2_ represents the response portion related to the 2nd order modal parameters ω_2_ and r_2_, and E_1_, E_2_ are constants representing the participation degrees of Y_ω1_ and Y_ω2_ in total response y_o_(t). Y_ω1_, Y_ω2_, E_1_ and E_2_ can be expressed as:

(16) $$Y_{\omega 1}=Y_{1R}\sin\left(\left(\omega_{d}+\omega \right)t+\phi_{1R}\right)+Y_{1L}\sin\left(\left(\omega_{d}-\omega \right)t+\phi_{1L}\right)$$

(17) $$Y_{\omega 2}=Y_{2R}\sin\left(\left(\omega_{d}+\omega \right)t+\phi_{2R}\right)+Y_{2L}\sin\left(\left(\omega_{d}-\omega \right)t+\phi_{2L}\right)$$

(18) $$\begin{array}{cc} E_{1}=\frac{\left(1-r_{1}\right)\left(m_{1}A_{d1}-r_{1}m_{2}A_{d2}\right)}{m_{1}+{r_{1}}^{2}m_{2}}, & E_{2}=\frac{\left(1-r_{2}\right)\left(m_{1}A_{d1}-r_{2}m_{2}A_{d2}\right)}{m_{1}+{r_{2}}^{2}m_{2}} \end{array}$$

where Y_1R_, Y_1L_, Y_2R_, Y_2L_ and ϕ_1R_, ϕ_1L_, ϕ_2R_, ϕ_2L_ can be expressed as:

(19) $$\begin{array}{cc} Y_{iR}=\frac{\omega_{d}}{\sqrt{{\left[{\omega_{i}}^{2}-{\left(\omega_{d}+\omega \right)}^{2}\right]}^{2}+{\left[2\xi_{i}\omega_{i}\left(\omega_{d}+\omega \right)\right]}^{2}}}, & \phi_{iR}=-\arctan\frac{2\xi_{i}\omega_{i}\left(\omega_{d}+\omega \right)}{{\omega_{i}}^{2}-{\left(\omega_{d}+\omega \right)}^{2}},i=1,2 \end{array}$$

(20) $$Y_{iL}=\frac{\omega_{d}}{\sqrt{{\left[{\omega_{i}}^{2}-{\left(\omega_{d}-\omega \right)}^{2}\right]}^{2}+{\left[2\xi_{i}\omega_{i}\left(\omega_{d}-\omega \right)\right]}^{2}}},\phi_{iL}=-\arctan\frac{2\xi_{i}\omega_{i}\left(\omega_{d}-\omega \right)}{{\omega_{i}}^{2}-{\left(\omega_{d}-\omega \right)}^{2}},i=1,2$$

Our analysis is based on the condition that frequency difference exists between the anti-phase mode in drive direction and that in sense direction, and the modal damping ratios are rather small. Therefore, the normalized drive velocity signal sin(ω_d_t) was used as the demodulation reference signal. After demodulation and low pass filtering, the final output voltage signal would be:

(21) $$v_{o}\left(t\right)=k_{vy}\Omega \cdot \left\{\frac{E_{1}}{2}\left[Y_{1R}\cos\left(\omega t+\phi_{1R}\right)+Y_{1L}\cos\left(\omega t-\phi_{1L}\right)\right]+\frac{E_{2}}{2}\left[Y_{2R}\cos\left(\omega t+\phi_{2R}\right)+Y_{2L}\cos\left(\omega t-\phi_{2L}\right)\right]\right\}$$

where k_vy_ is the overall gain from sense displacement y_o_ to sense voltage v_o_. Noted that only the mechanical part of the tuning fork micro-gyroscope is brought into discussion in this paper, hence the frequency response of the sensing circuits is ignored here.

Summation of signals with the same frequency was conducted on Equation (21). The relationship between the output voltage signal and the frequency of the input angular rate was obtained as follows:

(22) $$v_{o}\left(t\right)=k_{vy}\Omega \cdot \frac{Y_{o}}{2}\cos\left(\omega t+\varphi_{o}\right)$$

(23) $$Y_{o}\left(\omega \right)=\sqrt{\begin{array}{l} {\left[E_{1}\left(Y_{1R}\cos\phi_{1R}+Y_{1L}\cos\phi_{1L}\right)+E_{2}\left(Y_{2R}\cos\phi_{2R}+Y_{2L}\cos\phi_{2L}\right)\right]}^{2}\\ +{\left[E_{1}\left(Y_{1R}\sin\phi_{1R}-Y_{1L}\sin\phi_{1L}\right)+E_{2}\left(Y_{2R}\sin\phi_{2R}-Y_{2L}\sin\phi_{2L}\right)\right]}^{2} \end{array}}$$

(24) $$\varphi_{o}\left(\omega \right)=\arctan\frac{E_{1}\left(Y_{1R}\sin\phi_{1R}-Y_{1L}\sin\phi_{1L}\right)+E_{2}\left(Y_{2R}\sin\phi_{2R}-Y_{2L}\sin\phi_{2L}\right)}{E_{1}\left(Y_{1R}\cos\phi_{1R}+Y_{1L}\cos\phi_{1L}\right)+E_{2}\left(Y_{2R}\cos\phi_{2R}+Y_{2L}\cos\phi_{2L}\right)}$$

From Equations (19), (20), (23) and (24), it can be found that the amplitude-frequency curve Y_o_(ω) would have four peak points when E_1_ and E_2_ are all non-zero. The four peck frequencies are:

(25) $$\begin{array}{cccc} \omega_{r11}=\left|\omega_{1}-\omega_{d}\right|, & \omega_{r12}=\omega_{1}+\omega_{d}, & \omega_{r21}=\left|\omega_{2}-\omega_{d}\right|, & \omega_{r22}=\omega_{2}+\omega_{d} \end{array}$$

### 3.2. Simplified Equation in Low Frequency Range

The working frequency of a tuning fork micro-gyroscope is generally several kilohertz, and the necessary bandwidth is normally less than several hundred Hertz. Hence, the two peak points in high frequency range, ω_r12_ and ω_r22_, can be ignored in the discussion of bandwidth characteristics. In low frequency range, it can be considered that:

(26) $$\begin{array}{ccc} \omega \ll \omega_{d}, & \omega \ll \omega_{1}, & \omega \ll \omega_{2} \end{array}$$

For the convenience of analysis, we defined two sign parameters, s_1_ and s_2_, to identify the relative size of ω_1_, ω_2_ and ω_d_. The related expressions are shown as follows:

(27) $$\begin{array}{cc} s_{1}=\left\{ \begin{array}{l} \begin{array}{cc} +1\;, & \omega_{1}>\omega_{d} \end{array}\\ \begin{array}{cc} -1\;, & \omega_{1}<\omega_{d} \end{array} \end{array}\right., & s_{2}=\left\{ \begin{array}{l} \begin{array}{cc} +1\;, & \omega_{2}>\omega_{d} \end{array}\\ \begin{array}{cc} -1\;, & \omega_{2}<\omega_{d} \end{array} \end{array}\right. \end{array}$$

(28) $$\begin{array}{cccc} \Delta \omega_{1}=\left|\omega_{1}-\omega_{d}\right|, & \omega_{1}-\omega_{d}=s_{1}\Delta \omega_{1}, & \Delta \omega_{2}=\left|\omega_{2}-\omega_{d}\right|, & \omega_{2}-\omega_{d}=s_{2}\Delta \omega_{2} \end{array}$$

Two effective Q-factors, Q_e1_ and Q_e2_, were also defined based on the actual Q-factors Q_1_ and Q_2_:

(29) $$\begin{array}{cccc} Q_{e1}=\frac{\Delta \omega_{1}}{\omega_{1}}Q_{1}, & Q_{e2}=\frac{\Delta \omega_{2}}{\omega_{2}}Q_{2}, & Q_{1}=\frac{1}{2\xi_{1}}, & Q_{2}=\frac{1}{2\xi_{2}} \end{array}$$

Then in low frequency range, Equations (19) and (20) can be approximately simplified as:

(30) $$\begin{array}{cc} Y_{iR}\approx \frac{\omega_{d}}{\omega_{i}\Delta \omega_{i}\sqrt{4{\left(s_{i}-\frac{\omega }{\Delta \omega_{i}}\right)}^{2}+\frac{1}{{Q_{ei}}^{2}}}}, & \tan\phi_{iR}\approx \frac{-1/Q_{ei}}{2\left(s_{i}-\frac{\omega }{\Delta \omega_{i}}\right)},i=1,2 \end{array}$$

(31) $$\begin{array}{cc} Y_{iL}\approx \frac{\omega_{d}}{\omega_{i}\Delta \omega_{i}\sqrt{4{\left(s_{i}+\frac{\omega }{\Delta \omega_{i}}\right)}^{2}+\frac{1}{{Q_{ei}}^{2}}}}, & \tan\phi_{iL}\approx \frac{-1/Q_{ei}}{2\left(s_{i}+\frac{\omega }{\Delta \omega_{i}}\right)},i=1,2 \end{array}$$

While Q_i_ is rather large and Δω_i_ is not very small, it can be approximately considered that:

(32) $$\{\begin{array}{ccc} \cos\phi_{iR}\approx -1, & \sin\phi_{iR}\approx 0, & s_{i}=-1\\ \cos\phi_{iL}\approx +1, & \sin\phi_{iL}\approx 0, & s_{i}=+1 \end{array},i=1,2$$

By this time, if we define new parameters Y_iP_, Y_iS_, and ϕ_iP_ as:

(33) $$Y_{iP}\approx \frac{\omega_{d}}{\omega_{i}\Delta \omega_{i}\sqrt{4{\left(1-\frac{\omega }{\Delta \omega_{i}}\right)}^{2}+\frac{1}{{Q_{ei}}^{2}}}},Y_{iS}\approx \frac{\omega_{d}}{\omega_{i}\Delta \omega_{i}\sqrt{4{\left(1+\frac{\omega }{\Delta \omega_{i}}\right)}^{2}+\frac{1}{{Q_{ei}}^{2}}}},i=1,2$$

(34) $$\cos\phi_{iP}\approx \frac{2\left(1-\frac{\omega }{\Delta \omega_{i}}\right)}{\sqrt{4{\left(1-\frac{\omega }{\Delta \omega_{i}}\right)}^{2}+\frac{1}{{Q_{ei}}^{2}}}},\sin\phi_{iP}\approx \frac{-\frac{1}{Q_{ei}}}{\sqrt{4{\left(1-\frac{\omega }{\Delta \omega_{i}}\right)}^{2}+\frac{1}{{Q_{ei}}^{2}}}},i=1,2$$

where subscript P represents the primary portion closely related to the peak points, and subscript S represents the subordinate portion unrelated to the peak points, then in low frequency range, Equations (22)–(24), representing the relationship between the output voltage signal and the frequency of the input angular rate, can be simplified as:

(35) $$v_{o}\left(t\right)=k_{vy}\Omega \cdot \frac{Y_{o}}{2}\cos\left(\omega t+\varphi_{o}\right)$$

(36) $$Y_{o}\left(\omega \right)\approx \sqrt{{\left[E_{1}s_{1}\left(Y_{1P}\cos\phi_{1P}+Y_{1S}\right)+E_{2}s_{2}\left(Y_{2P}\cos\phi_{2P}+Y_{2S}\right)\right]}^{2}+{\left[E_{1}s_{1}\left(Y_{1P}\sin\phi_{1P}\right)+E_{2}s_{2}\left(Y_{2P}\sin\phi_{2P}\right)\right]}^{2}}$$

(37) $$\varphi_{o}\left(\omega \right)\approx \arctan\frac{E_{1}s_{1}\left(Y_{1P}\sin\phi_{1P}\right)+E_{2}s_{2}\left(Y_{2P}\sin\phi_{2P}\right)}{E_{1}s_{1}\left(Y_{1P}\cos\phi_{1P}+Y_{1S}\right)+E_{2}s_{2}\left(Y_{2P}\cos\phi_{2P}+Y_{2S}\right)}$$

## 4. Bandwidth Characteristics under Ideal Conditions

Under ideal conditions, the structure of a tuning fork micro-gyroscope is full symmetric, and the drive amplitudes are also the same with AGC loop control, that is:

(38) $$\begin{array}{ccc} k_{11}=k_{22}=k_{0}, & m_{1}=m_{2}=m_{0}, & A_{d1}=A_{d2}=A_{d0} \end{array}$$

In this condition, it can be calculated from Equations (10) and (11) that the amplitude ratio r_1_ would be +1 and r_2_ would be −1. That is, in 1st and 2nd order modal vectors, the two sense masses present in-phase and anti-phase mode vibrations with the same amplitude respectively. With Equation (18), the participation degrees were obtained as:

(39) $$\begin{array}{cc} E_{1}=0, & E_{2}=2A_{d0} \end{array}$$

Equation (39) indicates that under ideal conditions, the 1st vibration mode, *i.e.*, the in-phase vibration mode, would not participate in anti-phase Coriolis forces induced vibration response. With Equations (35), (36), (38) and (39), the input-output amplitude-frequency response under ideal conditions can be found as:

(40) $$\frac{V_{o}\left(\omega \right)}{\Omega }\approx k_{vy}\cdot A_{d0}\sqrt{{Y_{2P}}^{2}+{Y_{2S}}^{2}+2Y_{2P}Y_{2S}\cos\phi_{2P}}$$

where V_o_ represents the amplitude of v_o_(t). By substituting Equations (33) and (34) into Equation (40), the specific expression would be:

(41) $$\frac{V_{o}\left(\omega \right)}{\Omega }\approx \frac{k_{vy}A_{d0}\cdot \omega_{d}}{\omega_{2}\Delta \omega_{2}\sqrt{4{\left(1-\frac{\omega }{\Delta \omega_{2}}\right)}^{2}+\frac{1}{{Q_{e2}}^{2}}}}\sqrt{1+\frac{4{\left(1-\frac{\omega }{\Delta \omega_{2}}\right)}^{2}+\frac{1}{{Q_{e2}}^{2}}}{4{\left(1+\frac{\omega }{\Delta \omega_{2}}\right)}^{2}+\frac{1}{{Q_{e2}}^{2}}}+\frac{4\left(1-\frac{\omega }{\Delta \omega_{2}}\right)}{\sqrt{4{\left(1+\frac{\omega }{\Delta \omega_{2}}\right)}^{2}+\frac{1}{{Q_{e2}}^{2}}}}}$$

Equation (41) is similar to a resonant system with a peak frequency of Δω_2_ and an effective Q-factor of Q_e2_. The peak amplitude and the initial amplitude have the relationship of V_o_(Δω_2_)/V_o_(0) ≈ Q_e2_.

Actually, as only the anti-phase mode participates in the sense mode vibration response, under ideal conditions, the input-output amplitude-frequency response of a tuning fork micro-gyroscope is equivalent to that of a single mass micro-gyroscope with a sense mode natural frequency of ω_2_. Equation (41) has the same form with that of a single mass micro-gyroscope [11].

**Figure 3.**
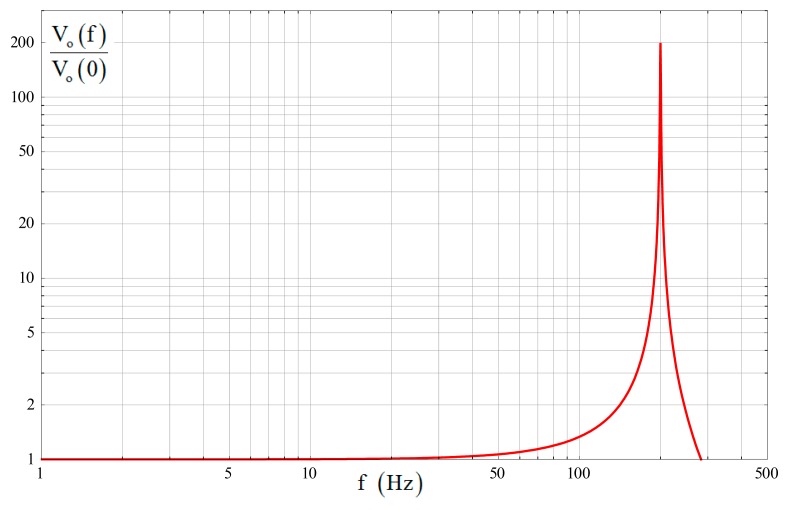
Input-output amplitude-frequency response under ideal conditions.

A tuning fork micro-gyroscope with f_d_ = 1800 Hz, f_2_ = 2000 Hz, Δf_2_ = 200 Hz, Q_2_ = 2000 and Q_e2_ = 200 was taken as an example to show the bandwidth characteristics under ideal conditions. Here f denotes the frequency value corresponding to each angular frequency. The amplitude-frequency curve generated by Equation (41) is shown in Figure 3. It is well consistent with the previous analysis.

There are two cases of the gyroscope 3-dB bandwidth depending on Q_e2_. With a relatively high Q_e2_, the peak amplitude at Δω_2_ will exceed the 3-dB scope and the gyroscope bandwidth would be smaller than Δω_2_. With a relatively low Q_e2_, the peak amplitude at Δω_2_ will be included in the 3-dB scope and the gyroscope bandwidth would be larger than Δω_2_.

While the frequency of the input angular rate ω is not very close to Δω_2_, the influence of Q_e2_ can be locally ignored and Equation (41) can be further simplified as:

(42) $$\frac{V_{o}\left(\omega \right)}{\Omega }\approx \frac{k_{vy}A_{d0}\omega_{d}}{\omega_{2}\Delta \omega_{2}}\cdot \frac{1}{\left|1-\frac{\omega }{\Delta \omega_{2}}\right|\left(1+\frac{\omega }{\Delta \omega_{2}}\right)}$$

By Equation (42), the two cases of gyroscope 3-dB bandwidth, with BW_H_ denoting the bandwidth with a high Q_e2_ and BW_L_ denoting the bandwidth with a low Q_e2_, were obtained as follows:

(43) $$\begin{array}{cc} BW_{H}\approx \Delta \omega_{2}\sqrt{1-\frac{1}{\sqrt{2}}}\approx 0.54\Delta \omega_{2}, & BW_{L}\approx \Delta \omega_{2}\sqrt{1+\sqrt{2}}\approx 1.55\Delta \omega_{2} \end{array}$$

## 5. Bandwidth Characteristics under Nonideal Conditions

In layout design, the structure of a tuning fork micro-gyroscope is generally fully symmetric. However, because of the unavoidable non-ideal factors such as fabrication errors, the actual structure is commonly asymmetric. For the bandwidth characteristics, our discussion covered three potentially harmful kinds of asymmetry: asymmetric drive amplitude, asymmetric sense mass and asymmetric sense stiffness.

### 5.1. Asymmetric Drive Amplitude

Asymmetric drive amplitude is mainly caused by the comb asymmetry in drive mechanisms of the two single mass structures. According to Equation (18), its influence on the frequency response is mainly from the participation degrees E_1_ and E_2_. Assuming that all other parameters remain symmetric, when A_d1_ ≠ A_d2_, the participation degrees would be:

(44) $$\begin{array}{cc} E_{1}=0, & E_{2}=A_{d1}+A_{d2} \end{array}$$

It is obvious that the in-phase vibration mode still would not participate in the anti-phase Coriolis forces induced vibration response. With Equations (35), (36) and (44), the input-output amplitude-frequency response in the presence of asymmetric drive amplitude would be:

(45) $$\frac{V_{o}\left(\omega \right)}{\Omega }\approx k_{vy}\cdot \frac{A_{d1}+A_{d2}}{2}\sqrt{{Y_{2P}}^{2}+{Y_{2S}}^{2}+2Y_{2P}Y_{2S}\cos\phi_{2P}}$$

Comparing Equation (45) with Equation (40), it can be found that when all other parameters remain symmetric, the asymmetric drive amplitude would only make a difference on the overall amplitude level of the input-output amplitude-frequency response.

### 5.2. Asymmetric Sense Mass and Stiffness

Asymmetric sense mass and stiffness have a direct influence on the natural frequencies and modal vectors of the 2-DOF sense mode vibration system. From Equations (10), (11) and (18), it can be found that in the presence of asymmetric sense mass and stiffness, the amplitude ratios r_1_ and r_2_ would deviate from +1 and −1, and the participation degrees E_1_ and E_2_ would be both non-zero. That is, both the in-phase vibration mode and the anti-phase one would participate in anti-phase Coriolis forces induced vibration response.

A tuning fork micro-gyroscope with design parameters given in Table 1 was taken as an example. Sense mode coupling method Type-B shown in Figure 2 was employed and the corresponding stiffness k_11_, k_22_ and k_12_ in Equation (9) would be:

(46) $$k_{11}=\frac{2k_{S1}\left(k_{S2}+k_{C}\right)}{k_{S1}+k_{S2}+k_{C}}\;,\;k_{22}=\frac{2k_{S2}\left(k_{S1}+k_{C}\right)}{k_{S1}+k_{S2}+k_{C}}\;,\;k_{12}=\frac{-2k_{S1}k_{S2}}{k_{S1}+k_{S2}+k_{C}}$$

To analyze the specific influence of the asymmetric sense mass and stiffness, we defined mass asymmetric coefficient α and stiffness asymmetric coefficient β as follows:

(47) $$\begin{array}{cc} m_{2}=\alpha m_{1}, & k_{S2}=\beta k_{S1} \end{array}$$

**Figure 4.**
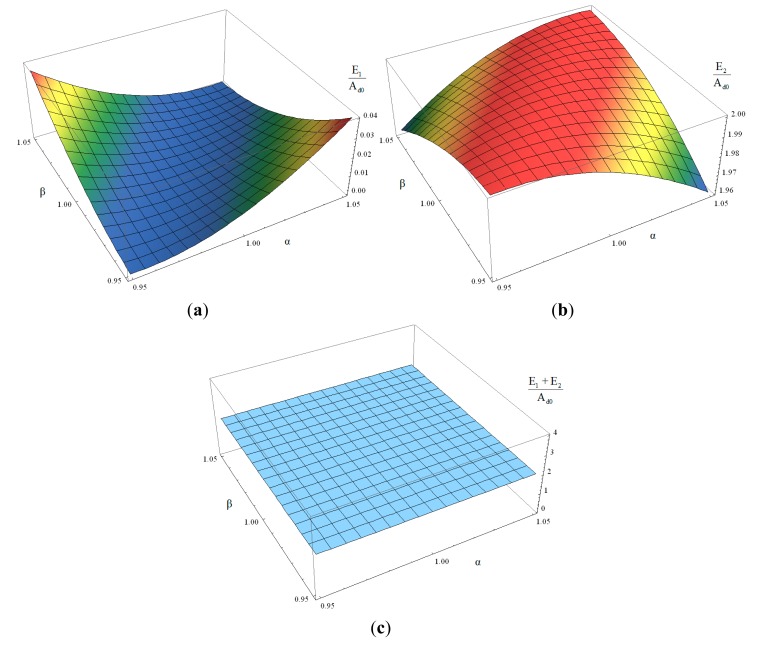
(**a**) Change of E_1_/A_d0_ with the variation of α and β; (**b**) Change of E_2_/A_d0_ with the variation of α and β; (**c**) Change of (E_1_ + E_2_)/A_d0_ with the variation of α and β.

With the assumption of symmetric drive amplitude, *i.e.*, A_d1_=A_d2_=A_d0_, change of the participation degrees E_1_ and E_2_ with the variation of α and β can be obtained from Equations (10), (11), (18), (46) and (47). While α and β change in a ±5% variation range, *i.e.*, from 0.95 to 1.05 respectively, the change tendencies of E_1_ and E_2_ are shown in Figure 4.

It can be found that while α and β change with the equal proportion in same direction, participation degrees E_1_ and E_2_ would always be 0 and 2A_d0_; while α and β change with different proportions, E_1_ would be larger than 0 and E_2_ would be smaller than 2A_d0_; while α and β reach their extreme values in opposite direction, E_1_ would get its maximum value and E_2_ would get its minimum one. No matter how α and β change, the sum of E_1_ and E_2_ would always be 2A_d0_.

It is obvious that while mass asymmetric coefficient α and stiffness asymmetric coefficient β change with different proportions, both the in-phase vibration mode and the anti-phase one would participate in anti-phase Coriolis forces induced vibration response. In this case, according to Equations (35) and (36), the input-output amplitude-frequency response in low frequency range would be:

(48) $$\frac{V_{o}\left(\omega \right)}{\Omega }\approx \frac{k_{vy}}{2}\sqrt{\begin{array}{c} {\left[E_{1}s_{1}\left(Y_{1P}\cos\phi_{1P}+Y_{1S}\right)+E_{2}s_{2}\left(Y_{2P}\cos\phi_{2P}+Y_{2S}\right)\right]}^{2}\\ +{\left[E_{1}s_{1}\left(Y_{1P}\sin\phi_{1P}\right)+E_{2}s_{2}\left(Y_{2P}\sin\phi_{2P}\right)\right]}^{2} \end{array}}$$

**Figure 5.**
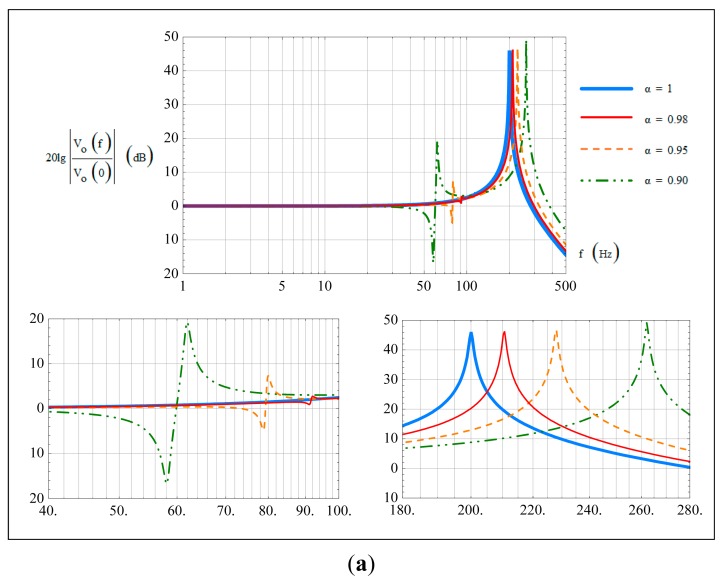

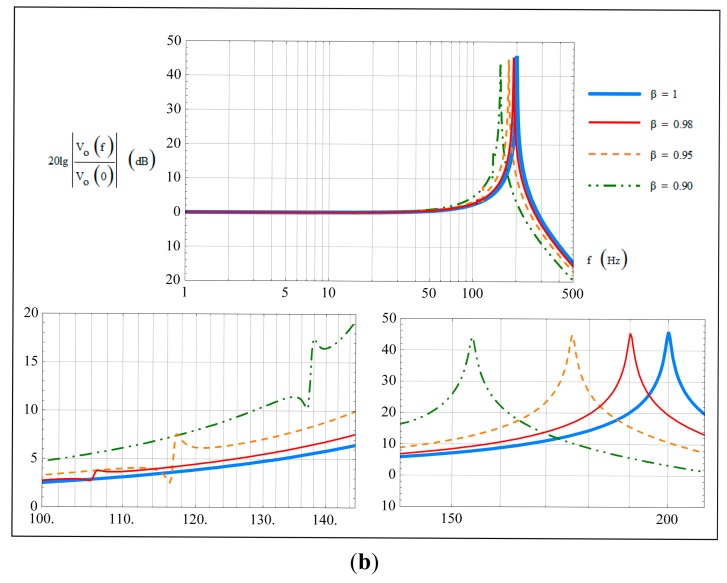
(**a**) Input-output amplitude-frequency response with non-ideal α values; (**b**) Input-output amplitude-frequency response with non-ideal β values.

Two cases were considered in our investigation to show the specific influence of non-ideal α and β on the bandwidth characteristics. In the first case, sense stiffness was assumed to be symmetric, *i.e.*, β = 1, and a comparison of the amplitude-frequency curves with α being 1, 0.98, 0.95 and 0.90 are shown in Figure 5a. In the second case, sense mass was assumed to be symmetric, *i.e.*, α = 1, and a comparison of the amplitude-frequency curves with β being 1, 0.98, 0.95 and 0.90 are shown in Figure 5b. The corresponding changes of the natural frequencies f_1_, f_2_ and the frequency differences Δf_1_, Δf_2_ in these two cases are shown in Table 2.

In the first case, *i.e.*, β=1, with decreasing mass asymmetric coefficient α, the natural frequencies f_1_ and f_2_ both increase. For f_d_ was designed between f_1_ and f_2_ as shown in Table 1, the frequency difference Δf_1_ decreases and Δf_2_ increases. Compared Figure 5a with Table 2, it can be found that when α < 1, the input-output amplitude-frequency curves would have two peak points, with peak frequencies locating at Δf_1_ and Δf_2_. The fluctuation phenomenon appears around Δf_1_, with a fluctuant extent increasing with the degree of asymmetry.

In the second case, *i.e.*, α = 1, with decreasing stiffness asymmetric coefficient β, the natural frequencies f_1_ and f_2_ both decrease, hence the frequency difference Δf_1_ increases and Δf_2_ decreases. Comparing Figure 5b with Table 2, it can be found that when β < 1, the input-output amplitude-frequency curves also have two peak points, with peak frequencies locating at Δf_1_ and Δf_2_. Similarly, fluctuation phenomenon appears around Δf_1_, with a fluctuant extent increasing with the degree of asymmetry.

In fact, for the anti-directional impact of α and β on E_1_ and E_2_, the input-output amplitude-frequency curves with β > 1 are similar to those with α < 1, and the input-output amplitude-frequency curves with α > 1 are similar to those with β < 1.

Under ideal conditions, the gyroscope 3-dB bandwidth is decided only by the amplitude variation around Δf_2_, and the mechanical bandwidth design is commonly based on this frequency difference. However, according to the previous analysis, under non-ideal conditions, the gyroscope 3-dB bandwidth may also be disturbed by the amplitude variation around Δf_1_. Especially in the case that Δf_1_ < Δf_2_, and the degree of mass and/or stiffness asymmetry is large enough to cause the fluctuant extent around Δf_1_ exceeding the 3-dB scope, the actual bandwidth would be much smaller than the commonly designed one.

## 6. Experimental Results

To verify the correctness of the theoretical analyses, two tuning fork micro-gyroscope prototypes designed in our laboratory, referred to as SG-1 and SG-2, were taken into input-output amplitude-frequency tests using a virtual rate-table method [12]. These two prototypes have the same mechanical structure form, but different natural frequency configurations. They were fabricated and packaged in different research stages and the natural frequency configuration of SG-2 is better than that of SG-1.

**Figure 6.**
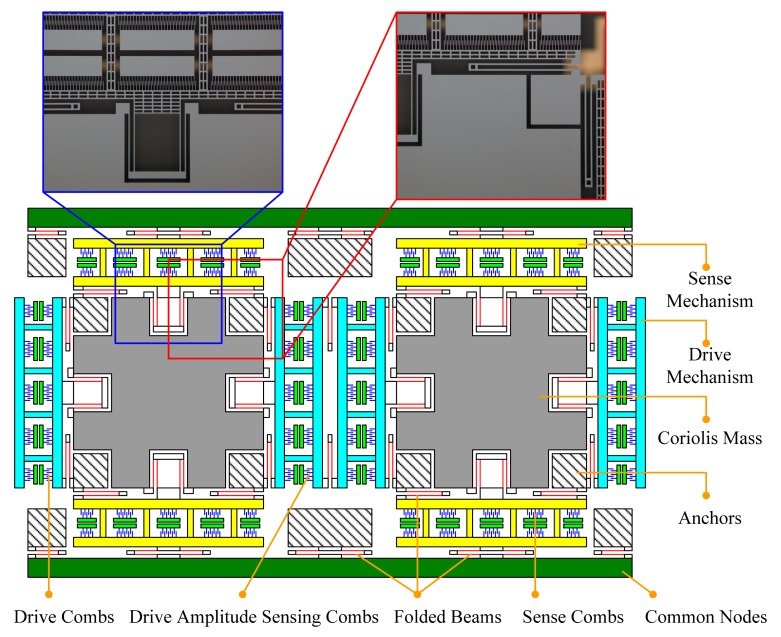
Mechanical structure of the designed tuning fork micro-gyroscope.

**Figure 7.**
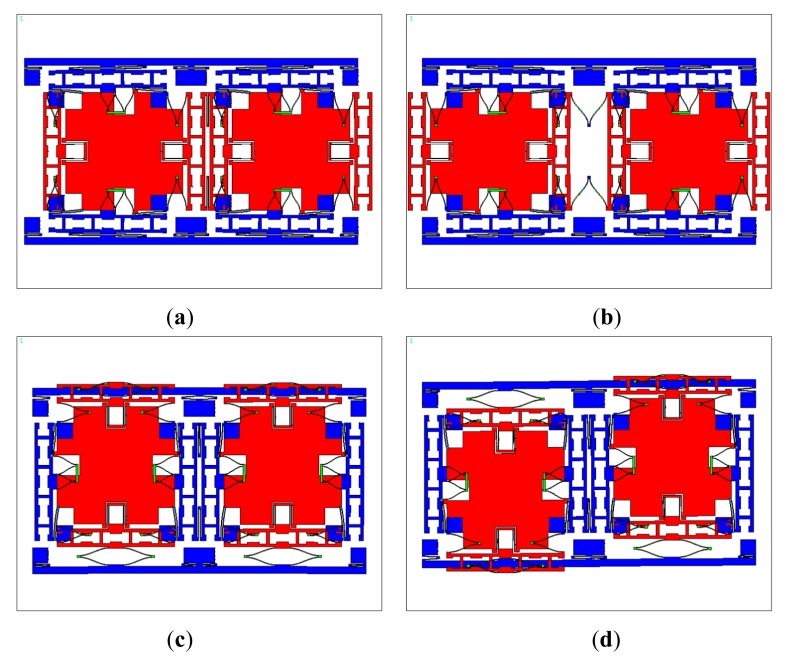
(**a**) In-phase mode pattern in drive direction; (**b**) Anti-phase mode pattern in drive direction; (**c**) In-phase mode pattern in sense direction; (**d**) Anti-phase mode pattern in sense direction.

**Figure 8.**
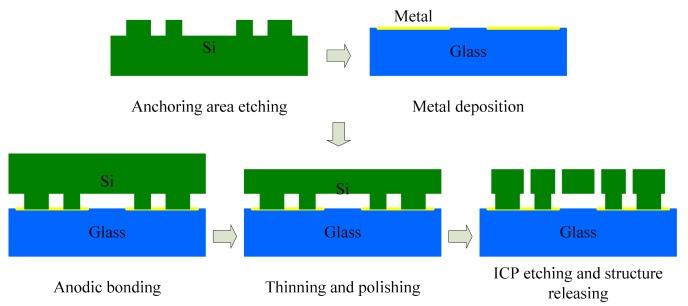
Fabrication process of Deep Dry Silicon On Glass (DDSOG).

The designed tuning fork micro-gyroscope is shown in Figure 6. Each single mass structure is fully decoupled, that is, 1-DOF drive mechanism and 1-DOF sense mechanism were designed along with the 2-DOF Coriolis mass. Both drive mode and sense mode are mechanically coupled. Drive mode mechanical coupling was realized with two folded beams. Sense mode mechanical coupling was realized with the application of coupling method Type-B shown in Figure 2. With the help of the finite element analysis software ANSYS, we got the four in-plane vibration mode patterns: in-phase mode in drive direction, anti-phase mode in drive direction, in-phase mode in sense direction and anti-phase mode in sense direction, which are shown in Figure 7. In the discussion of bandwidth characteristics, we put the main focus on the natural frequencies of the later three mode patterns. The mechanical structures were fabricated by the Deep Dry Silicon On Glass (DDSOG) process. The procedure of this fabrication process is shown in Figure 8.

**Figure 9.**
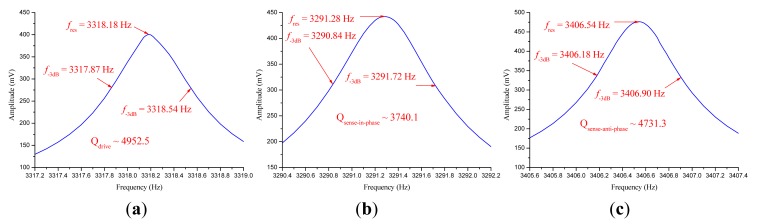
(**a**) The modal spectrum of anti-phase mode in drive direction in SG-1; (**b**) The modal spectrum of in-phase mode in sense direction in SG-1; (**c**) The modal spectrum of anti-phase mode in sense direction in SG-1.

**Figure 10.**
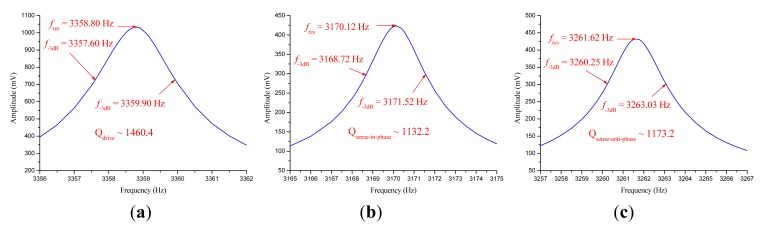
(**a**) The modal spectrum of anti-phase mode in drive direction in SG-2; (**b**) The modal spectrum of in-phase mode in sense direction in SG-2; (**c**) The modal spectrum of anti-phase mode in sense direction in SG-2.

Both prototypes were vacuum packaged with ceramic packages. The measured parameters of the two prototypes after vacuum packaging are shown in Table 3. In prototype SG-1, the natural frequencies f_d_, f_1_ and f_2_, respectively, corresponding to anti-phase mode in drive direction, in-phase mode in sense direction and anti-phase mode in sense direction, were configured as f_1_ < f_d_ < f_2_. In prototype SG-2, the natural frequencies were configured as f_1_ < f_2_ < f_d_. The modal spectra indicating resonant frequencies and quality factors of these three modes in SG-1 and SG-2 are shown in Figures 9 and 10, respectively.

**Figure 11.**
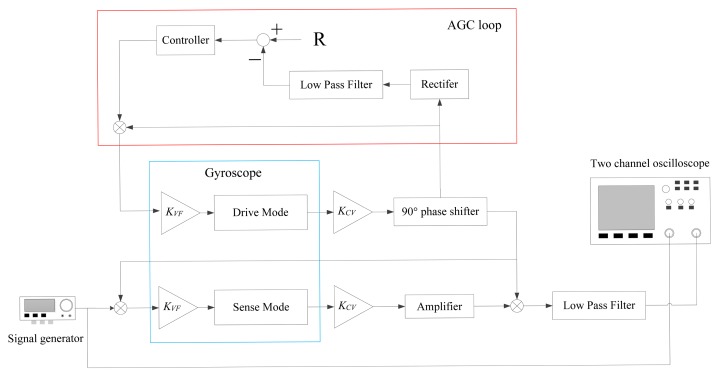
The functional block diagram of virtual rate-table method.

The virtual rate-table method was used to get the input-output amplitude-frequency response of the two prototypes. The functional block diagram of this method is shown in Figure 11. In the experimental tests, the drive mode of the tuning fork micro-gyroscope was actuated into anti-phase resonance vibration with constant amplitude. A signal generator was used to provide a cosine signal of varying frequency imitating the dynamic input angular rate. We named this the virtual angular rate signal. The multiplication of the virtual angular rate signal and the drive velocity signal was used to imitate the Coriolis force. Two anti-phase Coriolis force signals from the left and right single mass structures of the tuning fork micro-gyroscope were then applied onto the sense combs to actuate the vibration of the sense mode in an anti-phase way. Corresponding to different frequencies of the input virtual angular rate signal, the differential output amplitude values of the sense mode vibration response were collected.

**Figure 12.**
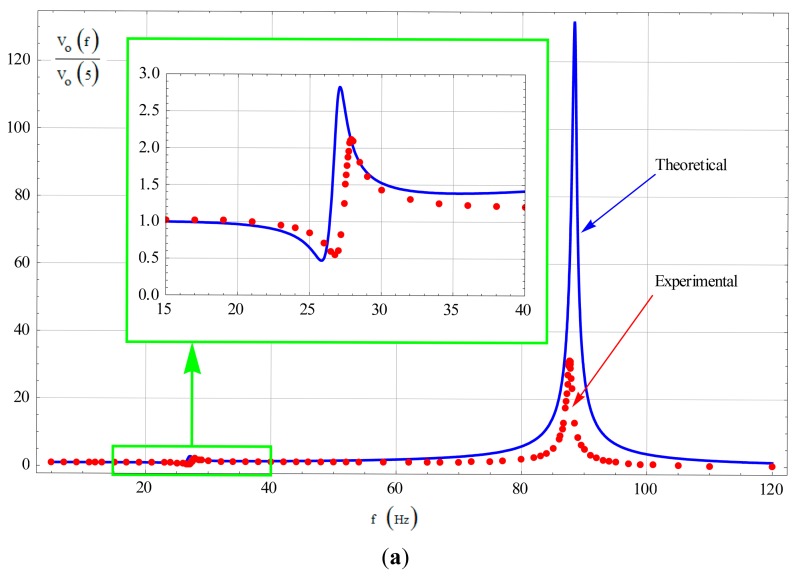

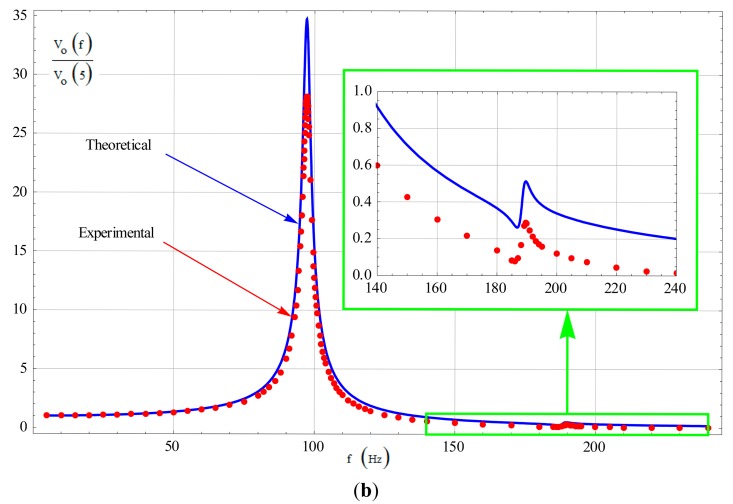
(**a**) Theoretical and experimental input-output amplitude-frequency responses of SG-1; (**b**) Theoretical and experimental input-output amplitude-frequency responses of SG-2.

In our designed tuning fork micro-gyroscope, the Coriolis mass is quite large and lots of flexure beams are used, hence the fabrication induced stiffness asymmetry is much more severe than the mass asymmetry. With the assumption of symmetric mass, the stiffness asymmetric coefficient β of prototype SG-1 was estimated to be 0.98, and the stiffness asymmetric coefficient β of prototype SG-2 was estimated to be 0.99. The theoretical amplitude-frequency curves were generated from Equation (48), and the experimental amplitude-frequency curves were obtained from tests. Comparison of the theoretical and experimental curves of SG-1 and SG-2 are shown in Figure 12a and Figure 6b, respectively.

It could be found that the theoretical curves coincide well with the experimental ones. Hence Equation (48) could be used to estimate the input-output amplitude-frequency response under nonideal conditions, providing a reference for mechanical bandwidth design. It is noted that in Figure 12, when the frequency of the input angular rate is relatively high, e.g., f > 70 Hz, the experimental results would be smaller than the theoretical ones in an overall level. That is mainly caused by the frequency response of the sensing circuits ignored in this paper. It is now undergoing further study.

It is obvious that the amplitude-frequency curves of the two prototypes, SG-1 and SG-2, both have two peak points for the unavoidable asymmetry caused by fabrication errors. The primary peak point locates at frequency difference Δf_2_, which is the main consideration in traditional bandwidth design. The subordinate peak point locates at frequency difference Δf_1_, which is non-ignorable in bandwidth design of a tuning fork micro-gyroscope with mechanically coupled sense mode.

In prototype SG-1, Δf_1_ is designed to be smaller than Δf_2_, hence when the degree of asymmetry is large enough to cause the fluctuant extent around Δf_1_ to exceed the 3-dB scope, and the gyroscope 3-dB bandwidth will be decided by the amplitude variation around Δf_1_ instead of Δf_2_. In prototype SG-2, Δf_1_ is designed to be larger than Δf_2_, hence no matter how large the degree of asymmetry is, as long as the frequency difference between Δf_1_ and Δf_2_ is large enough, the gyroscope 3-dB bandwidth will always be decided by the amplitude variation around Δf_2_. Therefore, to insure an appropriate bandwidth, the design value of Δf_1_ is recommended to be larger than Δf_2_, or at least make the key frequency point, where the maximum likelihood asymmetry induced fluctuant extent exceeding the 3-dB scope, to be larger than the necessary bandwidth.

Actually, the natural frequency configuration in prototype SG-2 (f_1_ < f_2_ < f_d_) is an improved version of that in prototype SG-1 (f_1_ < f_d_ < f_2_) based on the deduced equations and analytical results. Compared with the amplitude variation around Δf_1_ which is largely influenced by uncertain fabrication errors, the amplitude variation around Δf_2_ is much more stable. Therefore, it is strongly recommended that Δf_1_ = |f_1_ − f_d_| be larger than Δf_2_ = |f_2_ − f_d_| in mechanical design.

However, with a relatively low working frequency to improve the mechanical sensitivity, most of the time Δf = |f_2_ − f_1_| couldn't be large enough. Hence with the commonly seen natural frequency configuration in SG-1, when a large bandwidth is needed, Δf_2_ would have to be designed larger and Δf_1_ would be quite small. That would generate a disturbing fluctuation phenomenon in the useful bandwidth range and deteriorate the dynamic sensing capability of the micro-gyroscope. The improved natural frequency configuration in SG-2 can overcome this problem and has provided an application example of the deduced equations and analytical results.

## 7. Conclusions

Micro-gyroscopes need a certain bandwidth for dynamic angular rate sensing. For a tuning fork micro-gyroscope with mechanically coupled sense mode, in-phase and anti-phase vibration modes coexist in the sense direction. Under ideal (*i.e.*, symmetric) conditions, their bandwidth characteristics are similar to those of a single mass micro-gyroscope. The input-output amplitude-frequency curve has only one peak point, with a peak frequency locating at the frequency difference between the drive frequency and the natural frequency of the anti-phase mode in sense direction. The gyroscope 3-dB bandwidth is decided by the amplitude variation around this peak frequency only.

Under non-ideal (*i.e.*, asymmetric) conditions, the in-phase mode in the sense direction may participate in the anti-phase Coriolis forces induced vibration response, thus disturbing the bandwidth characteristics. The asymmetric drive amplitude would only make a difference on the overall amplitude level of the input-output amplitude-frequency curve, but the asymmetric sense mass and sense stiffness would have a direct influence on the natural frequencies and modal vectors of the 2-DOF sense mode vibration system. When the sense mass asymmetry and the sense stiffness asymmetry have equal proportions in same direction, the bandwidth characteristics would be the same as those under ideal conditions. When the sense mass asymmetry and the sense stiffness asymmetry have different proportions, the input-output amplitude-frequency curve would have two peak points. The primary peak point locates at the frequency difference between the drive frequency and the natural frequency of the anti-phase mode in the sense direction, which also exists under ideal conditions. The subordinate peak point locates at the frequency difference between the drive frequency and the natural frequency of the in-phase mode in the sense direction, which is caused by the participation of the in-phase mode in the sense vibration response. In this condition, the gyroscope 3-dB bandwidth would be decided by the amplitude variation around these two peak frequencies synthetically.

Conclusively, in the mechanical bandwidth design of a tuning fork micro-gyroscope with mechanically coupled sense mode, the relationships between the drive frequency and the natural frequencies of the in-phase mode and the anti-phase mode in sense direction, as well as the maximum likelihood asymmetry should all be taken into account. The equations representing the relationships between the differential output signal and the frequency of the input angular rate were deduced theoretically and verified by experimental results. They can be used as references in mechanical bandwidth design of a tuning fork micro-gyroscope with mechanically coupled sense mode.

## Figures and Tables

**Table 1.** Design parameters of the tuning fork micro-gyroscope taken as an example.

**Symbols**	**Descriptions**	**Design Values**
k_S1_	Sense stiffness of the left structure	39.48 N/m
k_S2_	Sense stiffness of the right structure	39.48 N/m
k_C_	Coupling stiffness	205.58 N/m
m_1_	Sense mass of the left structure	0.5 × 10^−6^ Kg
m_2_	Sense mass of the right structure	0.5 × 10^−6^ Kg
f_d_	Drive frequency	1800 Hz
f_1_	Natural frequency of the 1st order sense mode	1700 Hz
f_2_	Natural frequency of the 2nd order sense mode	2000 Hz
Q_1_	Q-factor of the 1st order sense mode	1800
Q_2_	Q-factor of the 2nd order sense mode	2000

**Table 2.** Change of the natural frequencies with nonideal α or β values.

**Natural frequencies (Hz)**	**β = 1**			**α = 1**		
	
	**α = 0.98**	**α = 0.95**	**α = 0.90**	**β = 0.98**	**β = 0.95**	**β = 0.90**
f_1_	1708.4	1720.2	1738.1	1693.6	1683.0	1662.4
f_2_	2010.5	2027.9	2062.1	1990.2	1976.0	1954.0
Δf_1_ = |f_1_−f_d_|	91.6	79.8	61.9	106.4	117.0	137.6
Δf_2_ = |f_2_−f_d_|	210.5	227.9	262.1	190.2	176.0	154.0

**Table 3.** Measured parameters of the two tuning fork micro-gyroscope prototypes.

**Measured Parameters**	**SG-1**	**SG-2**
f_d_	3318.2 Hz	3358.8 Hz

f_1_	3291.3 Hz	3170.1 Hz
Δf_1_ = |f_1_ − f_d_|	26.9 Hz	188.7 Hz
s_1_	−1	−1
Q_1_	3740.1	1132.2

f_2_	3406.5 Hz	3261.6 Hz
Δf_2_ = |f_2_ − f_d_|	88.3 Hz	97.2 Hz
s_2_	+1	−1
Q_2_	4731.3	1173.2
